# Positive causal association between diabetes and osteomyelitis, mediated by glycosylated hemoglobin and BMI: Evidence from a Mendelian randomization study

**DOI:** 10.1097/MD.0000000000041688

**Published:** 2025-03-07

**Authors:** Ke Zhang, Maosen Geng, Peiwu Zhang

**Affiliations:** a Department of Orthopedic, Xi’an Central Hospital, Xi’an, Shaanxi, China.

**Keywords:** body mass index, diabetes, glycosylated hemoglobin, mediation Mendelian randomization, osteomyelitis, two-sample Mendelian randomization

## Abstract

Diabetes mellitus (DM), a prevalent metabolic disorder, is intricately linked to various infectious diseases. Notably, osteomyelitis (OM), an infection affecting bone tissue, exhibits a higher incidence in individuals with DM. The primary objective of this study was to establish the causal association between DM and OM through Mendelian randomization (MR) analysis while also investigating potential mediating factors that may contribute to this relationship. The study utilized the two-sample Mendelian randomization (TSMR) approach to establish a causal link between type 1 diabetes (T1D), type 2 diabetes (T2D), and OM. The necessary data were obtained from a genome-wide association study, Data on T1D and T2D were obtained from FinnGen Biobank Round 5 Analysis (FINN) and the European Bioinformatics Institute (EBI). In TSMR, the primary analytical method chosen was inverse variance weighting. Additionally, mediation MR analysis was conducted to investigate potential mediators such as glycosylated hemoglobin (HbA1c), fructosamine, and body mass index (BMI). Results of TSMR analysis suggest a positive causal correlation between DM and OM, with DM increasing the risk of OM (T2D(FINN) on OM: odds ratio (OR) = 1.389 95%, confidence interval (CI): 1.215–1.588, *P* < .001. T2D(EBI) on OM: OR = 1.217 95%, CI: 1.007–1.470, *P* < .042) and T1D(FINN) on OM: OR = 1.140, 95% CI: 1.005–1.293, *P* = .042. T1D(EBI) on OM: OR = 1.261, 95% CI: 1.072–1.483, *P* < .005. Mediation MR results revealed that HbA1c and BMI act as facilitative mediators in the correlation between DM and OM. HbA1c in T1D-OM: OR = 1.379, 95% CI: 1.027–1.853, *P* < .001, and BMI in T1D-OM: OR = 1.691, 95% CI: 1.300–2.203, *P* < .001. HbA1c in T2D-OM: OR = 1.752, 95% CI: 1.290–2.377, *P* < .001, BMI in T2D-OM: OR = 1.788, 95% CI: 1.408–2.267, *P* < .001. The findings of this Mendelian randomization study provide evidence for a positive causal association between both 2 types of DM and OM in a European population. Subsequent mediation analysis revealed that HbA1c and BMI played a mediating role in this relationship.

## 1. Introduction

Osteomyelitis (OM) is an infectious bone inflammation that can be classified as either acute or chronic in nature. Symptoms commonly associated with acute OM include pain, fever, and localized swelling. The development of osteonecrosis typically does not occur within a few days to weeks following the initial infection. Chronic OM, on the other hand, is characterized by prolonged infection lasting for months to years, resulting in progressive bone destruction, formation of skin-to-bone fistulas, and recurrent episodes of skin breakdown.^[1,2]^ The classification of OM can be based on the infection mechanism, distinguishing between hematogenous and non-hematogenous types. Hematogenous OM occurs when bacteria disseminate through the bloodstream to the epiphyses of adjacent joints. This particular form is most prevalent among children, elderly individuals, and immunocompromised patients. On the other hand, non-hematogenous OM typically arises from direct inoculation during surgery or trauma or as a consequence of persistent soft tissue and joint infections.^[1–3]^ All forms of OM manifest indications of bacterial infection, with methicillin-susceptible *Staphylococcus aureus* being the predominant pathogen.^[4]^ In addition to these environmental influences, genetic factors have emerged as significant determinants in the development of OM. Several research findings suggest that single nucleotide polymorphisms (SNPs) may play a role in the genetic basis of OM. Notably, genetic variants at Bax gene G(-248), interferon-gamma, interleukin-1beta, and toll-like receptor 2 have been identified as associated with heightened susceptibility to hematogenous or traumatic OM.^[5–7]^ Meanwhile, the identification of the cyclooxygenase-2 enzyme has implicated a potential association with an elevated susceptibility to post-traumatic OM in individuals of Chinese descent.^[8]^

The prevalence of diabetes mellitus (DM), a chronic endocrine metabolic disease, has been steadily increasing over the years due to various changes in human lifestyles resulting from socio-economic development.^[9,10]^ It is projected that the prevalence of DM will experience a significant increase from 2.8% to 4.4% over the course of the next 3 decades, accompanied by a staggering rise in the total number of cases, estimated to surge by approximately 195 million.^[11]^ The literature has extensively documented the impact of DM on susceptibility to and outcomes of infectious diseases.^[12,13]^ DM can be complicated by a variety of infections, with urinary tract infections being the most prevalent among them.^[14]^ Diabetic patients are at an increased risk of developing cutaneous and subcutaneous infections, such as folliculitis and postoperative wound infections, in comparison to their nondiabetic counterparts.^[15,16]^ Emerging evidence suggests a potential association between elevated blood glucose levels and increased susceptibility to bacterial infections.^[17]^

Conventional observational studies are subject to various constraints, such as confounding variables, ethical considerations, limitations in research funding and time availability, alongside numerous other factors. In the Mendelian randomization (MR) study, genetic variation was utilized as instrumental variables (IVs) to investigate the causal relationship between exposure conditions and outcome factors. Additionally, based on odds ratio (OR) and 95% confidence interval (CI), the exposure factors were categorized into risk and protective factors.^[18–20]^ Genetic variants selected as IVs are assigned to offspring individuals immediately during conception, following the principles of Mendelian inheritance. As genetic correlations with disease remain unaffected by factors such as environmental influences, socio-economic status, or individual behaviors, MR studies effectively mitigate potential confounding factors and reverse causation effects when investigating causality. Thus, MR analysis can be regarded as a method for examining causality under specific assumptions.^[19–22]^ The present study employed a two-sample Mendelian randomization (TSMR) analysis to ascertain the causal relationship between the 2 types of DM and OM. Subsequently, mediation MR was utilized to investigate potential mediators that may contribute to this association.

## 2. Methods

### 2.1. Two-sample Mendelian randomization

**Figure 1.**
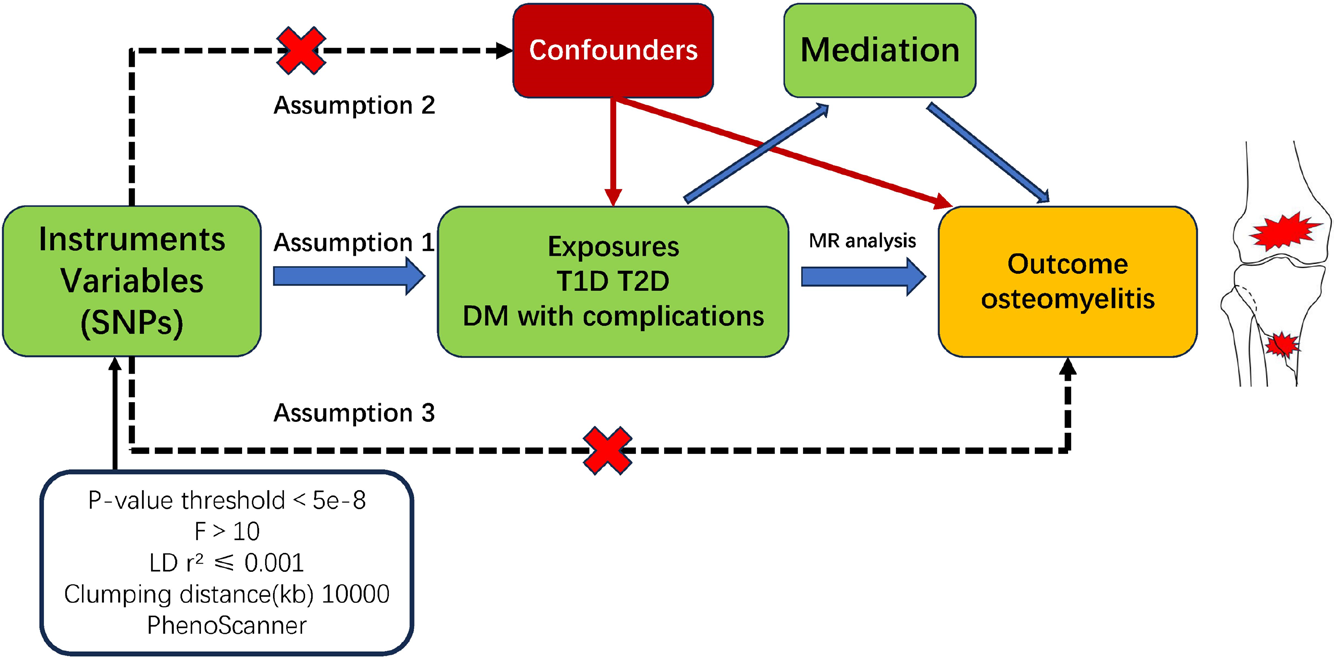
Study design and 3 basic assumptions.

In the TSMR study, type 1 diabetes (T1D), type 2 diabetes (T2D), and 2 types of DM with complications were considered as exposure factors, while OM served as the outcome factor. The study utilized SNPs identified from genome-wide association studies (GWAS), which were selected as IVs due to their strong associations with both DM and OM. The TSMR analysis adhered to 3 fundamental assumptions (Fig. 1): (1) IVs exhibited a robust correlation with the exposure factors; (2) IVs had no connections with potential confounders other than the exposure and outcome factors; (3) IVs solely influenced OM through DM without affecting the outcome via alternative pathways. The inverse variance weighting (IVW) method was utilized to assess the causal effects of T1D, T2D, and DM with complications on OM. Additionally, the analysis incorporated findings from weighted median, weighted mode, and MR-Egger methods. Heterogeneity of results was evaluated using Cochran *Q* test. A *P*-value > .05 indicates no significant heterogeneity among the variables under analysis. Pleiotropy detection employed both the MR-Pleiotropy Residual Sum and Outlier (MR-PRESSO) method as well as MR-Egger for assessment purposes. Absence of horizontal pleiotropy was determined if the *P*-value > .05. Furthermore, a leave-one-SNP-out analysis was conducted in this TSMR to further evaluate the reliability of the findings.

### 2.2. Mediation MR

By reviewing the existing literature on osteomyelitis risk factors, Massel DH et al identified high body mass index (BMI), smoking, and cortical bone erosion as potential risk factors for osteomyelitis.^[23]^ Previous studies have also demonstrated that glycosylated hemoglobin (HbA1c) and blood glucose levels can influence the balance between osteoblasts and osteoclasts.^[24–26]^ Considering abnormal clinical indicators in diabetic patients, we selected the following variables for analysis: anemia, daily cigarette consumption, fasting insulin levels, fructosamine levels, blood glucose levels, hypertension, renal or kidney issues other than diabetes-related complications (e.g., chronic kidney disease), C-reactive protein level, HbA1c level and BMI were included in mediation MR to explore their mediating effects between T1D, T2D, and osteomyelitis. The indirect effect of selected mediators was estimated using the 2-step MR approach.^[27]^ Firstly, the IV values of the exposure factors were utilized to estimate the causal effect of the exposure on the selected mediators. Secondly, the IV value of the mediator was employed to establish a causal relationship between intermediate factors and outcome measures. In mediation analysis, different mediators’ contributions were quantified by dividing their indirect effects by the total effect. Confidence intervals were estimated using the delta method.^[28]^

### 2.3. Data source

To minimize potential confounding bias resulting from racial stratification, our study exclusively focuses on individuals of European descent to ensure the reliability and consistency of our findings. Summary statistics for OM as an outcome factor were obtained from FinnGen Biobank Round 5 Analysis (FINN), which included a total of 359,772 participants of European descent (1758 cases and 358,014 controls). For more information about the database, please visit the official FINN website (https://www.finngen.fi/en). Additionally, we utilized 2 datasets from the Finnish database on T1D and T2D. These datasets comprised 4320 patients with T1D and 335,112 controls, as well as 65,085 patients with T2D and 335,112 controls respectively. SNPs associated with T1D and T2D were screened from 2 GWAS databases. Furthermore, we considered 2 other relevant databases related to DM: ebi-a-GCST90018925 (associated with T1D containing 6447 cases and 451,248 controls) and ebi-a-GCST006867 (associated with T2D containing 61,714 cases and 593,952 controls). Table 1 presents detailed information regarding the databases used in this study.

**Table 1:** Relevant information on the databases included in the analysis for this study.

GWAS ID	Phenotypes	Year	Author or consortium	Population	Sample size
					Cases controls
ebi-a-GST90018925	T1D	2021	Sakaue S	European	6447 451,248
finngen_R10_T 1D	T1D	2022	Finn	European	4320 335,112
finngen_R9_E4_DM1NASCOMP	T1D with complications	2022	Finn	European	6234 308,280
ebi-a-GST900167	T2D	2018	Xue A	European	61,714 593,952
finngen_R10_T2D	T2D	2022	Finn	European	65,085 335,112
finngen_R9_E4_DM2NASCOMP	T2D with complications	2022	Finn	European	46,373 308,280
ebi-a-GST90014006	HbA1c	2021	Mbatchou J	European	389,889
ieu-b-40	Body mass index	2018	GIANT	European	681,275
Finngen R10_M 13_OSTEOMYELITIS	OM	2022	Finn	European	1758 358,014

OM = osteomyelitis, T1D = type 1 diabetes, T2D = type 2 diabetes.

### 2.4. Selection of instrumental variables

The objective is to identify SNPs that exhibit strong associations with DM. As depicted in Figure 1, SNPs linked to both T1D and T2D underwent a rigorous screening process and were selected as IVs based on their statistical significance at *P* < 5e‐8. The analysis excluded SNPs with a linkage disequilibrium of *r*² ≤ 0.001 within a distance of 10,000 kb. This study utilized the European Reference Panel. Subsequently, we conducted F-statistics analysis to identify SNPs with F-values greater than or equal to 10, aiming to minimize potential analytical errors. The calculation of the F-value was based on the formula F = R2(N-2)/1-R2, where R2 = 2(1-MAF)*MAF*β^2 and N represents the total sample size. Here, β denotes the effect size of the exposure factor and MAF refers to minor allele frequency.^[29]^ The SNPs associated with OM were selected based on a statistical significance threshold of *P* < 5e‐5. However, SNPs exhibiting palindromic properties and intermediate allele frequencies were excluded from the analysis. To ensure the reliability of our findings and eliminate potential confounding variables, all exposure-independent SNPs that reached a statistical significance threshold of *P* < 5e‐5 were removed by cross-referencing the PhenoScanner database website.

### 2.5. MR analysis

The TSMR package (version 0.9.0) in the R software environment (version 4.3.3) was utilized to examine the causal relationship between T1D, T2D, and OM. This TSMR study employed several statistical methods including IVW, weighted median, weighted mode, and MR-Egger; with IVW being the principal analytical approach. Importantly, the IVW method is not limited by the assumption of weak IVs, thereby facilitating the generation of more robust estimation outcomes. MR-Egger regression can identify and adjust for pleiotropy. When at least 50% of the instrumental variables meet the criteria, employing a weighted median rule yields a more accurate conclusion. The MR estimates were reported as OR with corresponding 95% CIs. Exposure factors were considered as risk factors for the outcome when the OR exceeded 1, and conversely, exposure factors were regarded as protective factors for the outcome when the OR was <1. Conversely, in mediation analysis, an OR >1 indicates a facilitating effect of the mediator on exposure to the outcome, while a value <1 suggests an inhibiting effect.

## 3. Results

**Figure 2.**
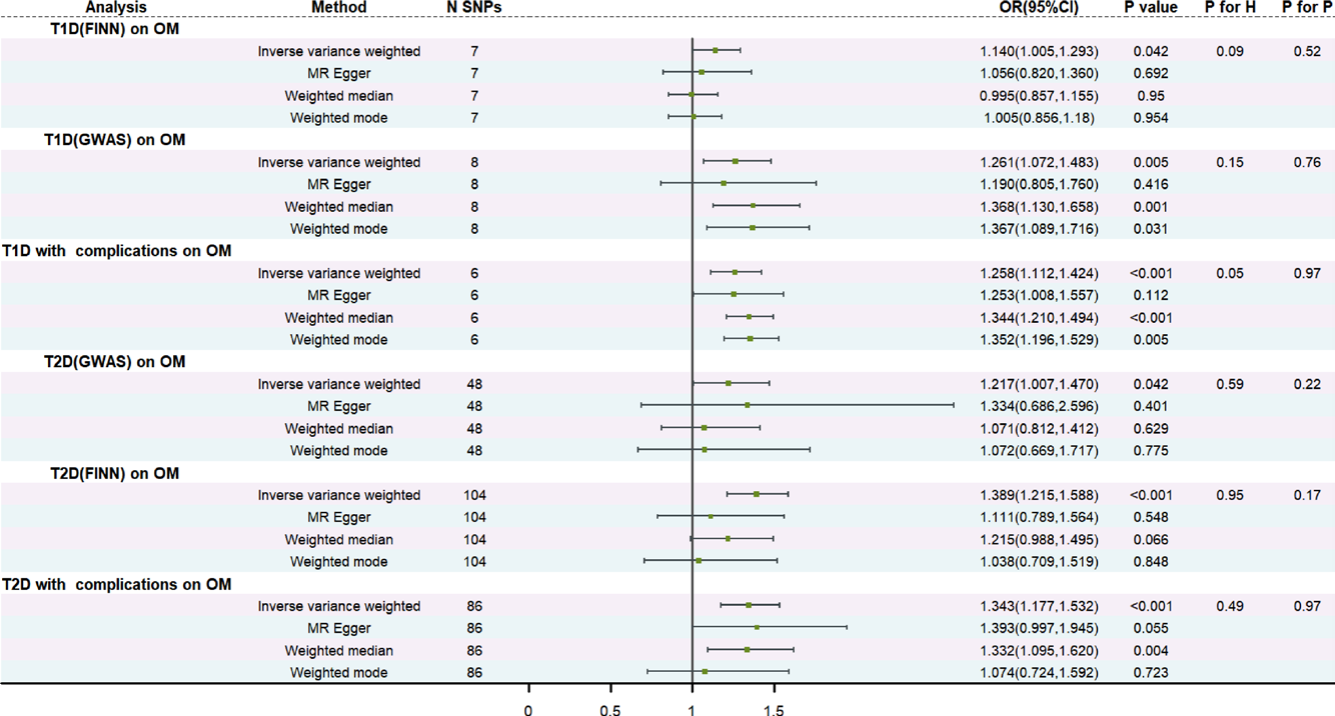
Genetically predicted the correlations between T1D, T2D, 2 types of DM with complications, and OM. “IVW,” “H,” “P,” “CI,” and “OR” stand for inverse variance weighted, heterogeneity, pleiotropy, confidence interval, and odds ratio. While a *P*-value < .05 was regarded as a statistically significant result. The FDR-corrected results of the *P*-values (IVW) in each subgroup remained consistent with the uncorrected results. DM = diabetes mellitus, OM = osteomyelitis, T1D = type 1 diabetes, T2D = type 2 diabetes.

**Figure 3.**
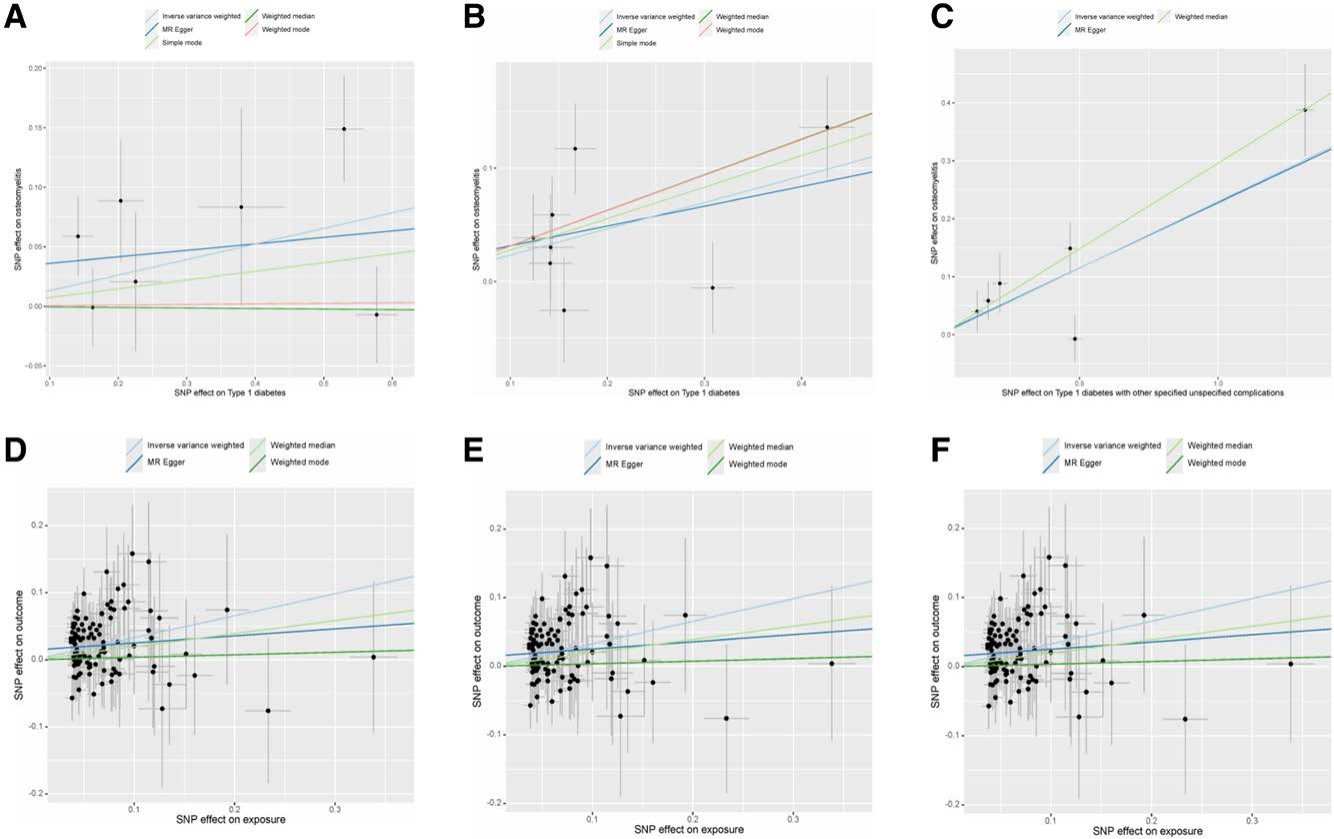
The scatter plot demonstrating the analysis of horizontal pleiotropy between T1D, T2D, 2 types of DM with complications, and OM. (A) T1D(FINN) on OM; (B) T1D(EBI) on OM; (C) T1D with complications on OM; (D) T2D(EBI) on OM; (E) T2D(FINN) on OM; and (F) T2D with complications on OM. DM = diabetes mellitus, EBI = European Bioinformatics Institute, FINN = FinnGen Biobank Round 5 Analysis, OM = osteomyelitis, T1D = type 1 diabetes, T2D = type 2 diabetes.

**Figure 4.**
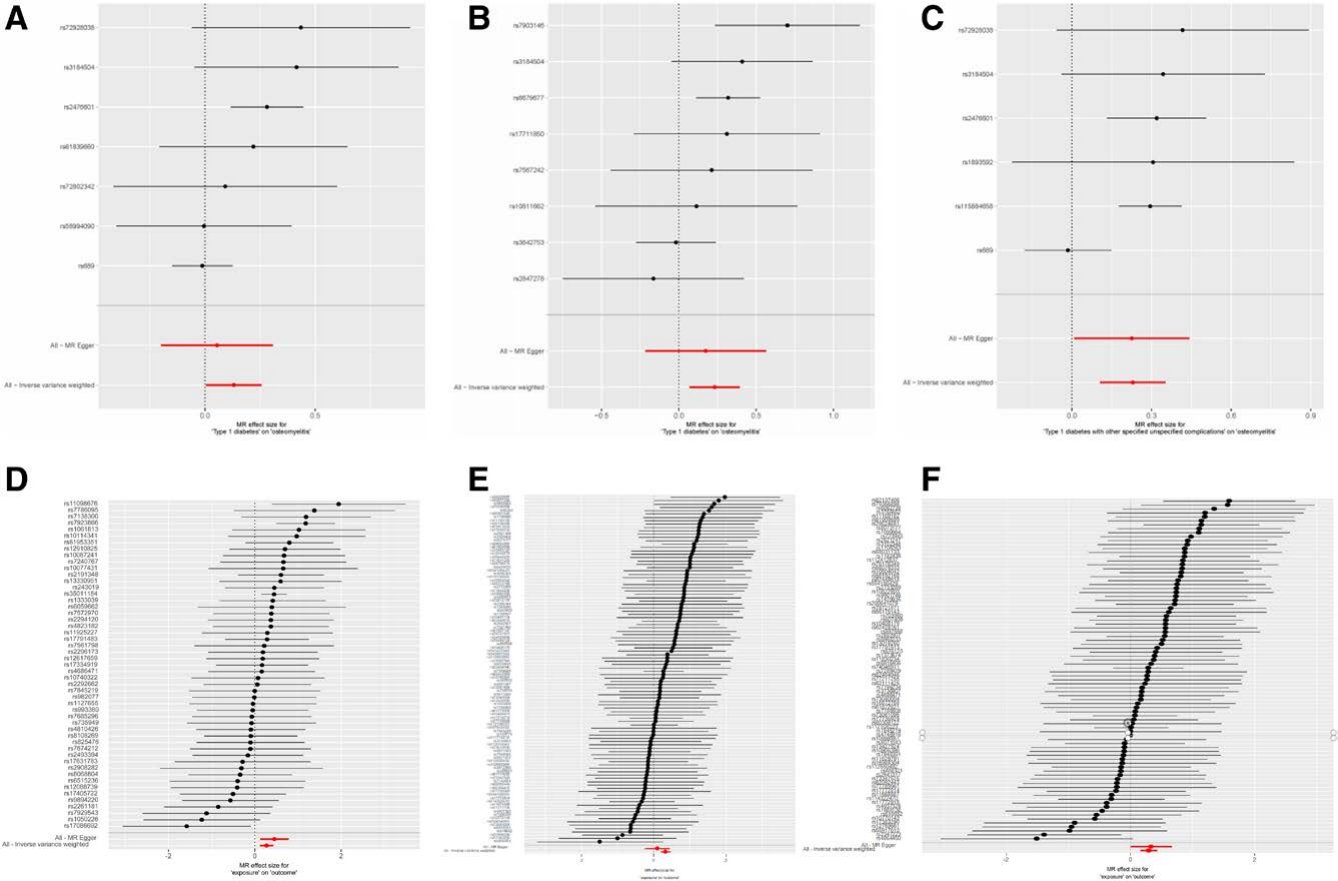
The results of leave-one-out analyses in the forest plot demonstrate that no SNPs could bias the results of the analysis between T1D, T2D, 2 types of diabetes with complications, and OM. (A) T1D(FINN) on OM; (B) T1D(GWAS) on OM; (C) T1D with complications on OM; (D) T2D(GWAS) on OM; (E) T2D(FINN) on OM; and (F) T2D with complications on OM. FINN, FinnGen Biobank Round 5 Analysis, GWAS = genome-wide association study, OM = osteomyelitis, SNP = single nucleotide polymorphisms, T1D = type 1 diabetes, T2D = type 2 diabetes.

**Figure 5.**
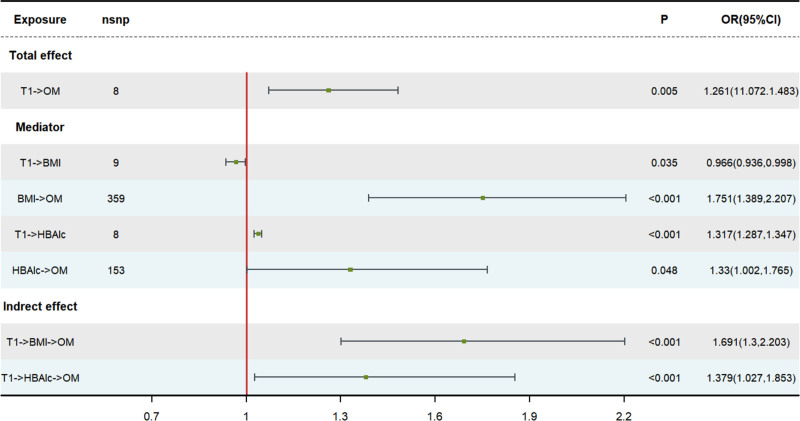
Results of a mediation MR analysis of T1D, T2D to OM by using a 2-step analysis. A *P*-value < .05 was regarded as a statistically significant result. The FDR-corrected results of the *P*-values (IVW) in each subgroup remained consistent with the uncorrected results. IVW = inverse variance weighting, OM = osteomyelitis, T1D = type 1 diabetes, T2D = type 2 diabetes.

**Figure 6.**
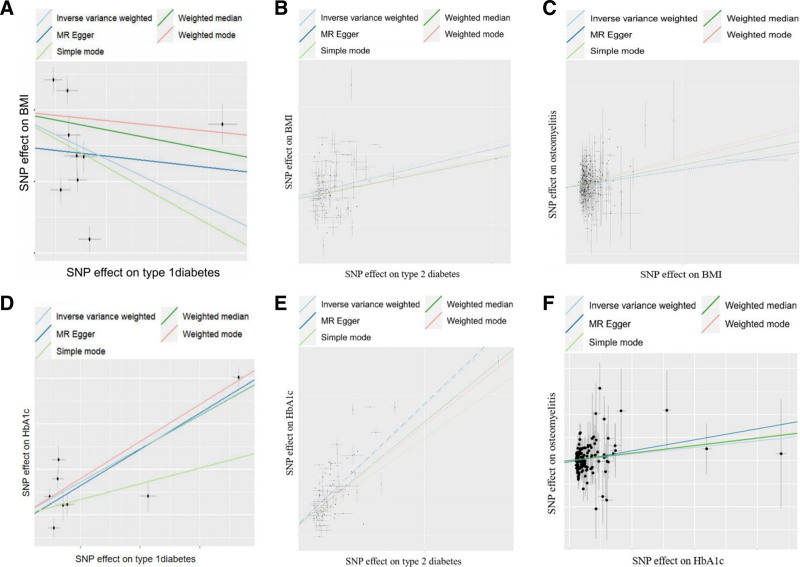
The scatter plot illustrates the horizontal pleiotropy analysis between the exposure factor and the mediator, as well as the mediator and OM. (A) T1D on BMI; (B) T2D on BMI; (C) BMI on OM; (D) T1D on HbA1c; (E) T2D on HbA1c; and (F) HbA1c on OM. BMI = body mass index, HbA1c = glycosylated hemoglobin, OM = osteomyelitis, T1D = type 1 diabetes, T2D = type 2 diabetes.

The results of the TSMR analysis revealed a statistically significant causal association between T1D, T2D, and OM (T1D to OM: OR = 1.140, 95% CI: 1.005–1.293, *P* = .042 and OR = 1.261, 95% CI: 1.072–1.483, *P* = .005. T2D to OM: OR = 1.389, 95% CI: 1.215–1.588, *P* < .001 and OR = 1.217, 95% CI: 1.007–1.470, *P* = .042). Additionally, both types of DM with complications exhibit a positive causal relationship with the occurrence of OM (T1D with complications to OM: OR = 1.258, 95% CI: 1.112–1.424, *P* < .001 T2D with complications to OM: OR = 1.343, 95% CI: 1.177–1.532, *P* < .001). The analysis findings did not reveal any significant heterogeneity or pleiotropy in the causal correlation between DM and OM (both heterogeneity and pleiotropy *P* > .05). Similarly, no evidence of pleiotropy or heterogeneity was observed in the correlation between DM with complications and OM. Comprehensive statistical data and visual representation regarding TSMR can be found in Figures 2–4. In our mediation analysis, we examined several potential mediators, including fructosamine, glucose levels, Hb1Ac, and BMI, to separately investigate their mediating effects on the associations between T1D and OM as well as T2D and OM. The results of 2-step mediator analysis provide evidence that both Hb1Ac and BMI serve as mediators in the causal relationships between these 2 types of DM and OM. The mediation role of HbA1c in the correlation between T1D and OM, as indicated by an odds ratio (OR) of 1.379 (95% CI: 1.027–1.853, *P* < .05), accounted for a mediated proportion of 5%. Similarly, the mediation role of HbA1c in the correlation between T2D and OM was supported by an OR of 1.752 (95% CI: 1.290–2.377, *P* < .05), with a mediated proportion of 28%. Furthermore, BMI also played a mediating role in the association between T1D and OM with an OR of 1.691 (95% CI: 1.300–2.203, *P* < .05) and a mediated proportion of 3%. Likewise, BMI acted as a mediator in the relationship between T2D and OM with an OR value of 1.788 (95% CI: 1.408–2.267, *P* < .05) and a mediated proportion of 4%. A visual representation illustrating these mediation effects is presented in Table 2, Figures 5 and 6. And the results of mediation MR analyses, including those that did not yield significant mediation effects, are summarized in Table S1, Supplemental Digital Content, http://links.lww.com/MD/O443.

**Table 2:** The mediation effect between OM and different types of diabetes through different mediating factors.

	Mediator	Total effect OR (95% CI)	Direct effect A OR (95% CI)	Direct effect B OR (95% CI)	Mediation effect OR (95% CI)	*P*	Mediated proportion(%)
T2D	HbA1c	1.31 (1.12, 1.54)	1.32 (1.29, 1.35)	1.33 (1.00, 1.77)	1.75 (1.29, 2.38)	<.05	28%
	BMI	1.31 (1.12, 1.54)	1.02 (1.01, 1.03)	1.75 (1.39, 2.20)	1.79 (1.41, 2.27)	<.05	4%
T1D	HbA1c	1.26 (1.07, 1.48)	1.04 (1.03, 1.05)	1.33 (1.00, 1.77)	1.38 (1.03, 1.85)	<.05	5%
	BMI	1.26 (1.07, 1.48)	0.97 (0.94, 0.99)	1.75 (1.39, 2.21)	1.691 (1.33, 2.20)	<.05	3%

BMI = body mass index, direct effect A = the influence of T1D and T2D on intermediary factors, direct effect B = the effect of intermediary factors on OM, HbA1c = glycosylated hemoglobin, mediation effect = the impact of T1D and T2D on osteomyelitis OM through various types of mediating factors, OM = osteomyelitis, T1D = type 1 diabetes, T2D = type 2 diabetes.

The total effect, direct effect A, and direct effect B were derived using the inverse variance weighted (IVW) method. The mediation effect was derived using the delta method. All statistical tests were conducted with a 2-sided hypothesis.

## 4. Discussion

The application of TSMR analysis in this study provides a genetic perspective to deduce a positive causal connection between 2 forms of DM with OM. Additionally, the mediation MR analyses conducted on potential mediators indicate that HbA1c and BMI act as intermediary factors in the causal association between DM and OM.

The pathogenesis of OM involves a bacterial infection that elicits an inflammatory response, leading to the deterioration of bone tissue. Acute OM predominantly affects immunocompromised individuals in the pediatric and geriatric populations, while chronic OM arises from surgical interventions or prolonged wound infections 1, 3. DM, a disease of the endocrine system, has emerged as one of the most prevalent and rapidly expanding global health issues due to socio-economic development and changes in population lifestyles. It is frequently accompanied by various complications such as nephropathy, diabetic retinopathy, and neuropathy. Additionally, DM can give rise to a range of infections including common urinary tract infections and soft tissue skin infections like cellulitis. Notably, one of the most prevalent complications associated with DM is the diabetic foot with an incidence rate ranging from approximately 12% to 25%. This condition imposes a significant financial burden on healthcare systems while also significantly reducing the quality of life for affected individuals.^[30]^ The incidence of postoperative incisional infections is higher in patients with DM. Several studies have indicated that elevated blood glucose levels are associated with an increased risk of bacterial infections.^[14–17]^ Several reports have suggested an elevated risk of OM development in diabetic patients compared to nondiabetic individuals.^[2,31,32]^ Nevertheless, the research on the correlation between DM and OM is constrained by several factors. Primarily, prospective clinical trials are essential to investigate this association; however, these trials face various restrictions including ethical considerations, study costs, and other confounding factors. To overcome these unfavorable circumstances, MR analysis was employed in this experiment to screen relevant SNPs as IVs from the GWAS database. Ultimately, the results of TSMR provide compelling evidence for a gene-predicted positive causal association between DM and OM. DM significantly increases the risk of OM, and this risk is further elevated in DM patients with complications. Therefore, it is imperative for individuals with DM to proactively manage their disease progression to mitigate the likelihood of developing complications and reduce the incidence of OM.

The regulation of the inflammatory response involves a diverse array of cytokines, which are considered pivotal mediators of inflammation. Several inflammatory mediators and cytokines, such as tumor necrosis factor-alpha, chemotaxis inhibitory proteins, and FPRL1 inhibitory protein, have been implicated in the pathogenesis of OM.^[33–36]^ The primary etiology of OM is attributed to Staphylococcus aureus, which produces adhesions facilitating its attachment to both cartilage and surgically implanted medical devices.^[4,37]^ A study investigated the regulatory role of Ddb1-cullin4-associated-factor 1 in protein ubiquitination within macrophages and its impact on OM.^[38]^ Additionally, a trend towards decreased expression and downregulation of peroxisome proliferator-activated receptor-γ (PPAR-γ) was observed in the infected bone tissue of patients with OM compared to those without OM. This suggests that PPAR-γ, as an anti-inflammatory factor, may play a role in modulating the inflammatory response through its regulation of osteoblast differentiation in the pathogenesis of OM.^[39]^

The correlation between HbA1c levels and an increased susceptibility to infectious diseases in patients with DM has been demonstrated by numerous studies. A significant correlation is believed to exist between elevated levels of glycated hemoglobin and urinary tract infections.^[40]^ A study demonstrates a potential association between Helicobacter pylori infection and HbA1c levels, with an additional finding that elevated HbA1c levels in individuals with DM are correlated with the severity of chronic gastritis.^[41,42]^ The relationship between HbA1c and bone health is believed to be linear. Hyperglycemia and HbA1c may impede the formation of osteoblasts from mesenchymal progenitors by activating the Notch2 signaling pathway.^[43]^ The accumulation of advanced glycation end products may result in a decrease in bone turnover, alterations in bone microarchitecture, and an elevated risk of fracture among diabetic patients.^[44–47]^

Obesity is a metabolic disease of great concern worldwide. Previous studies have shown that obesity (or higher BMI) is an important risk factor for some diseases. In terms of orthopedic diseases, obesity is associated with OM, osteopenia, and osteopenia.^[48–51]^ Furthermore, it is noteworthy that the impact of this phenomenon on bone metabolism can be attributed to intricate mechanisms involving both adipocytes and osteoblasts.^[52]^ The impact of palmitic acid, a fatty acid, on bone formation and osteoblast activity in obese animals has been demonstrated through the reduction of circulating markers.^[53]^ Bone morphogenetic proteins have also been implicated in the relationship between bone metabolism and obesity and glucose metabolism.^[54]^ Lipocalin signaling has also been implicated in the regulation of bone homeostasis, thereby potentially contributing to the pathogenesis of diseases such as osteogenesis.^[55]^ Our study provides evidence supporting a positive causal association between DM and OM at the genetic level, underscoring the significance of glycemic control and weight management in orthopedic patients. Particularly for obese individuals with T2D, effective body weight regulation, continuous blood glucose monitoring, and regular HbA1c assessments are instrumental in reducing postoperative OM incidence.

This study has the following strengths: Firstly, MR Analysis is used to prove that there is a positive causal relationship between DM and OM, and further mediating MR Shows that HbA1C and BMI play a mediating role in promoting the development of this causal relationship. This research method effectively avoids the influence of confounding factors such as external environment, acquired individual behavior, economic factors and social status, and has been widely used. Subsequently, we excluded all confounding SNPS except DM to ensure the robustness of the analysis. For DM of exposure factors, we selected genetic data from the 2 databases as instrumental variables (IVs), and obtained consistent conclusions, which reduced the sample overlap rate and further ensured the robustness of the conclusions. The findings of our study are in line with previous research and corroborated by our clinical expertise. However, there are still some limitations in this study. The GWAS data utilized in this study exclusively represents the European population, necessitating further confirmation to ascertain the generalizability of these findings across diverse ethnic groups. Given the constrained sample size of OM data, our pleiotropy test conducted during OM and DM TSWR failed to satisfy the Mendelian randomization hypothesis at a significance level lower than 0.05. As a result, we could not definitively establish a causal relationship between OM and DM. The mediating factors examined in this study were derived from the existing literature and integrated with our clinical expertise. However, it is important to acknowledge that there may exist additional potential mediating factors that were not included in the mediation analysis.

## 5. Conclusion

The findings from TSMR provide compelling evidence for a causal relationship between DM and OM. Specifically, the presence of DM as a risk factor significantly increases the susceptibility to OM in European populations. Mediation MR analysis suggests that both HbA1c and BMI may contribute to this association between DM and OM.

## Acknowledgments

We would like to express our sincerest gratitude to all the authors and participants in the GWAS study for providing the summary statistics used in this study.

## Author contributions

**Conceptualization:** Ke Zhang.

**Funding acquisition:** Peiwu Zhang.

**Investigation:** Ke Zhang.

**Methodology:** Ke Zhang, Maosen Geng, Peiwu Zhang.

**Project administration:** Ke Zhang, Peiwu Zhang.

**Software:** Maosen Geng.

**Visualization:** Maosen Geng.

**Writing – original draft:** Ke Zhang.

**Writing – review & editing:** Ke Zhang, Maosen Geng, Peiwu Zhang.

## Supplementary Material
