# Supplementary material for: Positive causal association between diabetes and osteomyelitis, mediated by glycosylated hemoglobin and BMI: Evidence from a Mendelian randomization study
Source: Medicine (Baltimore). 2025 Mar 7;104(10):e41688. doi: 10.1097/MD.0000000000041688 (PMC11902999; doi:10.1097/MD.0000000000041688)
Supplement: Supplementary file 1 [file medi-104-e41688-s001.docx]

Supplementary table 1. All mediation MR analyses results included in this study.

A two-step mediated MR Analysis (any step in the two-step method, P value > 0.05, that is, the intermediary effect does not exist, that is, the two-step method stops)
